# Identification of novel population clusters with different susceptibilities to type 2 diabetes and their impact on the prediction of diabetes

**DOI:** 10.1038/s41598-019-40058-y

**Published:** 2019-03-04

**Authors:** Seong Beom Cho, Sang Cheol Kim, Myung Guen Chung

**Affiliations:** Division of Biomedical Informatics, National Institute of Health, KCDC, Cheongju-si, Chungcheongbuk-do 28159 Republic of Korea

## Abstract

Type 2 diabetes is one of the subtypes of diabetes. However, previous studies have revealed its heterogeneous features. Here, we hypothesized that there would be heterogeneity in its development, resulting in higher susceptibility in some populations. We performed risk-factor based clustering (RFC), which is a hierarchical clustering of the population with profiles of five known risk factors for type 2 diabetes (age, gender, body mass index, hypertension, and family history of diabetes). The RFC identified six population clusters with significantly different prevalence rates of type 2 diabetes in the discovery data (*N* = 10,023), ranging from 0.09 to 0.44 (Chi-square test, *P* < 0.001). The machine learning method identified six clusters in the validation data (*N* = 215,083), which also showed the heterogeneity of prevalence between the clusters (*P* < 0.001). In addition to the prevalence of type 2 diabetes, the clusters showed different clinical features including biochemical profiles and prediction performance with the risk factors. SOur results seem to implicate a heterogeneous mechanism in the development of type 2 diabetes. These results will provide new insights for the development of more precise management strategy for type 2 diabetes.

## Introduction

Diabetes is one of the most prevalent chronic diseases. The affected population is still growing, and it is estimated that 4−5 million people will develop diabetes until 2030^1,2^. Considering socio-economic cost for life-long management of diabetes, identification of risk factors and preventing action is a crucial part of diabetes management, especially in terms of public health.

In clinical practice, diabetes is categorized according to a well-established classification system, which includes type 1, type 2, gestational, and other specific types of diabetes such as drug-induced diabetes and maturity-onset diabetes of the young^3–5^. Although type 2 diabetes is a primary element of the current classification system of diabetes, there is evidence of heterogeneity in type 2 diabetes^6^. For example, the glucose or insulin profile can also be used to stratify type 2 diabetes patients into groups with different prognoses^6^. In the research, type 2 diabetes patients showed a clear difference in glucose sensitivity and β-cell function according to the status of metabolic syndrome^6^. In addition to the heterogeneity of type 2 diabetes, there is heterogeneous effect of the risk factor for type 2 diabetes^7–15^. Here, the heterogeneity indicates different characteristics in the development of type 2 diabetes, For example, the association between waist circumference (WC) and type 2 diabetes was stronger in Australian population than in Iran population, which indicated the heterogeneous effect of WC in the type 2 diabetes between different ethnic groups^7^. In Japanese population, heterogeneous relationship of insulin resistance and development of type 2 diabetes was reported. They found that a small number of obese population showed insulin resistance at the beginning of type 2 diabetes, while most of population had decreased insulin secretion^8^. The heterogeneous effect of the risk factors is also associated with genetic effect for development of type 2 diabetes. In genome-wide association studies, stratification by sex, body mass index (BMI), or ethnic group shows heterogeneous associations with genetic loci in type 2 diabetes^9–16^. Genetic loci in the CDKAL1 and HHEX showed heterogeneous association with type 2 diabetes between gender group^9^. When type 2 diabetes groups was divided into obese and non-obese group, genetic loci that showed significant difference of effect size in the development of type 2 diabetes were found^10^. The heterogeneous genetic effect was found in the other studies with stratification of obesity^11–13^. The heterogeneous genetic effect was also found between different ethnic groups^14,15^. These results indicates that the risk factors for type 2 diabetes have heterogeneous effects that are related to different clinical characteristics or genetic effects in the development of diabetes.

**Figure 1:**
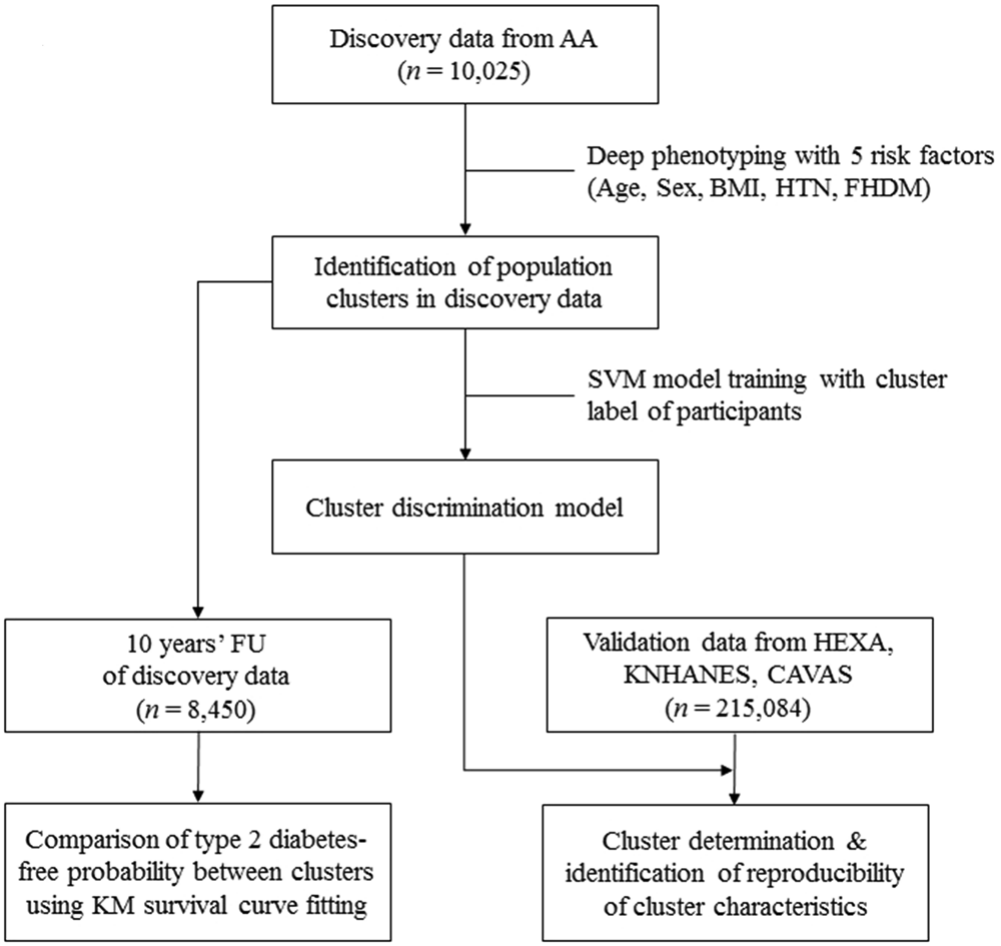
Study Design and Analysis. In this analysis, we used 4 cohort data sets for discovery and validation of population clusters with significantly different prevalence rates of type 2 diabetes. First, discovery data from the AA cohort (*n* = 10,023) were clustered into several groups until heterogeneity of the prevalence of type 2 diabetes was maximized. Next, we validated the reproducibility of the clusters using 2 validation data sets. The first validation data set included 10-year follow-up data for the participants who had not been diagnosed with diabetes in the discovery data (*n* = 7,574). We performed survival analysis using the disease-free time since diagnosis of type 2 diabetes to test whether the initial cluster was related to the future development of type 2 diabetes. The second validation data set included cross-sectional data that had been collected from 3 independent cohorts (HEXA, CAVAS, and KNHANES; *n* = 215,083). The reproducibility of cluster characteristics, in particular the prevalence of type 2 diabetes, were evaluated with the cross-sectional validation data. AA: Ansan/Ansung, BMI: body mass index, HTN: hypertension, FHDM: family history of diabetes, SVM: support vector machine, FU: follow-up, HEXA: Health Examinees study, KNHANES: Korea National Health and Nutrition Examination Survey, CAVAS: Cardiovascular Association Study, KM: Kaplan–Meier.

Previous research on the heterogeneity of type 2 diabetes is mostly based on stratification using a single risk factor. In this research, we hypothesized that stratification of type 2 diabetes based on multiple risk factors would show heterogeneity in the clinical features that cannot be identified from single-factor analysis. Although a previous study identified such clusters using combinations of categorized risk factors^16^, we identified the clusters using a data-driven method and validated the reproducibility of the heterogeneity in the independent data. For a proof-of-concept analysis, we applied machine learning technology to identify these heterogeneous clusters. First, we performed risk factor-based clustering (RFC) to identify population clusters with different prevalence rates of type 2 diabetes using hierarchical clustering based on risk factor profiles of individuals in the population. Here, the RFC indicates the application of clustering method to the phenotype profiles of risk factors using clustering method. We used the Ansan/Ansung cohort of Korea Genome and Epidemiology Study (KoGES) project^17^, which consists of suburban population in Republic of Korea, as the discovery data. After identifying the clusters in the discovery data, we compared the incidence rates of type 2 diabetes between clusters with those contained in a 10-year follow-up of the discovery data. We then validated the reproducibility of the difference between clusters in cross-sectional independent, large scale cohort data from Health Examinees (HEXA), and Cardiovascular Disease Association Study (CAVAS) of the KoGES project^17^. Figure 1 shows the overall analysis flow.

## Methods

### Study population

In total, 4 cohorts including those of the AA, HEXA, and CAVAS cohorts, which are part of the Korean Genome and Epidemiology Study cohort, and the Korea National Health and Nutrition Examination Survey (KNHANES) cohort^18^ were used. The detailed follow-up methods for AA, HEXA and CAVAS are described in previous report^17^. The KNHANES cohort recruit participants by visiting randomly selected areas of Republic of Korea. Until now, there is no follow-up for KNHANES. All participants were Korean and there were no other ethnic groups in the study.

The age, sex, and BMI information was obtained from the participants’ records. A family history of diabetes (FHDM) was identified from a questionnaire. If first degree relatives had any form of diabetes including type 1 and type 2, the FHDM was considered positive. Hypertension status (HTN) was determined based on medication history or current systolic pressure ≥140 mmHg or diastolic pressure ≥90 mmHg without diagnosis and treatment history. Type 2 diabetes was identified from a history of diagnosis or treatment of diabetes or according to the 2016 criteria of the American Diabetes Association: (1) fasting glucose concentration ≥126 mg/dL (7.0 mmol/L) or (2) glucose concentration ≥200 mg/dL (11.1 mmol/L) in 2-hour glucose from an oral glucose tolerance test (OGTT), or (3) HbA_1C_ level ≥6.5%. To rule out type 1 diabetes, we excluded participants whose age at diagnosis was <20 years. Participants with missing values in any of the risk factors were removed. Informed consent was obtained from all participants, and our institutional review board of National Institute of Health, KCDC approved this research. All data was gathered in accordance with Enforcement Decree of Bioethics and Safety Act, Republic of Korea. The KNHANES data can be downloaded from https://knhanes.cdc.go.kr. The KoGES data needs an application process (http://www.cdc.go.kr/CDC/contents/CdcKrContentView.jsp?cid=26458&viewType=CDC&menuIds=HOME001-MNU1136-MNU2530-MNU1223-MNU1542).

### RFC and construction of classification model for cluster discrimination

For RFC, we applied hierarchical clustering to the discovery data, which included information about the 5 risk factors for type 2 diabetes (age, sex, BMI, HTN, and FHDM). These risk factors are frequently used in the construction of prediction models for type 2 diabetes^19^. The dissimilarity between participants based on the profiles of the 5 risk factors was measured using the Gower distance because the risk factors contained both categorical and continuous variables^20^. Since the Gower distance standardized each variable and scaled the variables in the range of 0 to1, mixed variables can be applied simultaneously. The daisy function in R cluster package was used to compute the Gower distance^21^. Categorical data were coded as 0 or 1, which indicated the absence and presence of the status defined by the variables, respectively. Male and female participants were coded as 0 and 1. The computation method for binary variables was set to asymmetric, which indicates more weight on the presence of the risk factor in the computation of dissimilarity. The hclust function of R program was used for hierarchical clustering with the complete agglomeration method^22^. The cutree function was used to determine the cluster numbers. The cutree function is a basic function of R program, and it returns the cluster membership of instances according to the predefined number of clusters in the hierarchical clustering. Once the number of clusters is determined, the cutree function returns labels of the clusters of participants. Including the RFC, all statistical analyses were performed using R program (version 3.2.3)^22^.

The difference in the prevalence of type 2 diabetes between clusters was used as an indicator for stopping criterion in the determination of the number of clusters (=*k*). If the *k* is determined, the cutree function was applied and a 2 × *k* table was made according to the number of nondiabetic and diabetic subjects within each cluster. The heterogeneity of the 2 × *k* table was tested using Chi-square statistics. The Chi-square test was applied repeatedly by increasing the number of clusters by 1 each time, and the number of clusters was determined when the result of the Chi-square test with *k* clusters was more significant than that with *k* + 1 clusters.

After identifying the clusters in the discovery data, we developed the classification model to identify the clusters in the validation data. For this purpose, a support vector machine (SVM), which is a state-of-art classification machine learning algorithm, was used^23^. The dependent variable was set to cluster membership and the predictors were the 5 risk factors. The detailed procedure for building the classification model is described in Supplementary Methods. For estimation of area under curve (AUC), confidence interval (CI) and testing of difference between AUCs, we used pROC R package^24^.

## Results

### Discovery and validation cohort

In total, we used the 225,106 participants’ data from 4 different cohorts (AA, HEXA, CAVAS, and KNHANES, see Methods). The OGTT was applied in all follow-ups of the AA cohort, which allowed the accurate determination of type 2 diabetes. Therefore, we used the AA cohort as the discovery cohort. From the baseline investigation of the AA cohort, which was performed during 2001 and 2002, 10,023 participants without missing values were included (See Supplementary Results for data preprocessing).

After identification of the clusters, the reproducibility of the cluster was examined in the follow-up data of the AA cohort and the other 3 cohorts. The AA cohort is under biannual follow-up and we used the data that had been collected until the 5^th^ follow-up in 2011 and 2012^14^. The 8,450 participants who had no diabetes at the baseline investigation were included. Among them, the participants who had never been traced or had missing value in an OGTT or HbA_1C_ during follow-up were removed. A total of 7,574 participants and 61,880 person-years of follow-up were included. The average follow-up length was 8.17 person-years with standard deviation (SD) of 2.65 person-years, and the average number of visits per participants was 2.75 visits with SD of 1.58 visits. During the follow-up, 1,384 new cases of type 2 diabetes developed. The cross-sectional validation data (*N* = 215,083) comprised data from HEXA (*N* = 24,201), CAVAS (*N* = 162,773), and KNHANES (*N* = 28,109), which had no missing values for the 5 risk factors. For diabetes status, only the fasting glucose level and medical history were available in these 3 cohorts. The age range in the discovery data was 40–70 years; therefore, we included participants within this age range in the cross-sectional validation data. A summary of the 5 variables in the diabetes and nondiabetes groups is shown in Table 1.

**Table 1:** Summary of discovery and validation cohort.

Risk factor	Discovery cohort (AA, *n* = 10023)			Validation cohort (HEXA + CAVAS + KNHANES, *n* = 215083)		
	NDM (*n* = 8448)	DM (*n* = 1575)	*P* value	NDM (*n* = 194767)	DM (*n* = 20316)	*P* value
Sex (M/F)	3965/4483	787/788	0.03	67474/127293	9679/10637	*P* < 0.001
Age (mean ± SD)	51.59 ± 8.79	56.04 ± 8.76	*P* < 0.001	53.03 ± 8.31	57.59 ± 7.88	*P* < 0.001
BMI (mean ± SD)	24.42 ± 3.08	25.43 ± 3.34	*P* < 0.001	23.92 ± 2.93	24.98 ± 3.16	*P* < 0.001
HTN (−/+)	6242/2206	859/716	*P* < 0.001	146331/48436	8640/11676	*P* < 0.001
FHDM (−/+)	7062/846	1282/293	*P* < 0.001	163542/31225	14069/6247	*P* < 0.001

M: male, F: female, BMI: body mass index, HTN: hypertension, FHDM: family history of diabetes, NDM: non-type 2 diabetes, DM: type 2 diabetes, AA: Ansan/Ansung, HEXA: Health Examinees study, CAVAS: Cardiovascular Association Study, KNHANES: Korea National Health and Nutrition Examination Survey. Categorical and continuous data were compared using the Chi-square and *t* test, respectively.

### Risk factor-based clustering of the discovery data

Using RFC, we identified 6 clusters based on the heterogeneity of type 2 diabetes prevalence in the discovery data (Supplementary Results for determination process). Table 2 shows the summary statistics for the risk factors in each cluster. Dendrogram and heatmap are shown at Supplementary Fig. 2.

**Table 2:** Epidemiologic characteristics of clusters.

Cohort	Cluster (*n*)	Sex (M/F)	Age (mean ± SD)	BMI (mean ± SD)	HTN (−/+)	FHDM (−/+)	DM (−/+ prevalence)
Discovery (AA, *n* = 10023)	CL1 (1985)	M	45.72 ± 4.13	24.22 ± 2.81	(−)	(−)	1808/177 (0.09)^ǂ^
	CL2 (3241)	F	51.04 ± 8.74	24.35 ± 3.15	(−)	(−)	2937/304 (0.09)
	CL3 (862)	374/488	47.89 ± 6.90	24.61 ± 2.95	(−)	(+)	690/172 (0.20)
	CL4 (1013)	M	61.87 ± 4.23	23.11 ± 2.90	(−)	(−)	807/206 (0.20)
	CL5 (2645)	1258/1387	56.41 ± 8.57	25.50 ± 3.21	(+)	(−)	2050/595 (0.22)
	CL6 (277)	122/155	53.27 ± 8.45	26.20 ± 3.06	(+)	(+)	156/121 (0.44)
Validation (CAVAS HEXA KNHANES, *n* = 215083)	CL1 (24768)	M	46.84 ± 4.66	24.09 ± 2.88	(−)	(−)	23761/1007 (0.04)
	CL2 (83436)	F	51.90 ± 7.91	23.40 ± 2.85	(−)	(−)	80783/2653 (0.03)
	CL3 (27465)	8099/19366	50.55 ± 7.72	23.73 ± 2.88	(−)	(+)	24390/3075 (0.11)
	CL4 (19271)	M	61.70 ± 4.41	23.60 ± 2.62	(−)	(−)	17374/1897 (0.10)
	CL5 (50155)	21231/28924	57.33 ± 7.80	25.09 ± 3.06	31/50124^†^	50136/19^†^	41636/8519 (0.17)
	CL6 (9988)	3784/6204	55.50 ± 7.95	25.29 ± 3.03	(+)	(+)	6823/3165 (0.32)

M: male, F: female, BMI: body mass index, HTN: hypertension, FHDM: family history of diabetes, DM: diabetes, CL: cluster. AA: Ansan/Ansung, HEXA: Health Examinees study, CAVAS: Cardiovascular Association Study, KNHANES: Korea National Health and Nutrition Examination Survey.

^†^Although hypertension status and FHDM in cluster 5 show single results in discovery cohort, both statuses appear in cluster 5 of the validation cohort.

^ǂ^The prevalence is expressed to 2 digits after the decimal point.

The prevalence of type 2 diabetes differed significantly between the 6 clusters (Chi-square test, *P* = 3.16×10^−95^). We labeled the clusters according to the order of cluster-specific prevalence of type 2 diabetes (Table 2). The prevalence was the lowest (0.09) in cluster 1 (CL1) and highest (0.44) in cluster 6 (CL6), and had a 4.9-fold difference. The prevalence did not differ between CL1 and CL2, nor between CL3 and CL4, whereas when grouped together into 2 groups of clusters (CL1 and CL2 vs CL3 and CL4) the prevalence differed (0.09 vs 0.20, respectively). CL5 and CL6 showed a wider gap in prevalence (0.22 vs 0.44, respectively).

The clusters showed unique distributions of risk factors. The clusters tended to have a single category for each categorical risk factor. For example, HTN and FHDM had a single category of presence or absence within each cluster, whereas male and female sex appeared in the same cluster (Table 2). Each cluster showed a unique combination of risk factors. CL4 had the highest mean age, the lowest mean BMI and only males. Although CL1 and CL2 had similar prevalence, they showed clear differences in the distributions of sex and age. CL1 had only male and CL2 had only female participants, and the mean age was lower in CL1 (45.72 years) than in CL2 (51.04 years). CL6 had the highest prevalence of diabetes and the participants of the CL6 had the HTN, FHDM and highest mean BMI, but had an intermediate mean age (53.27 years).

**Figure 2:**
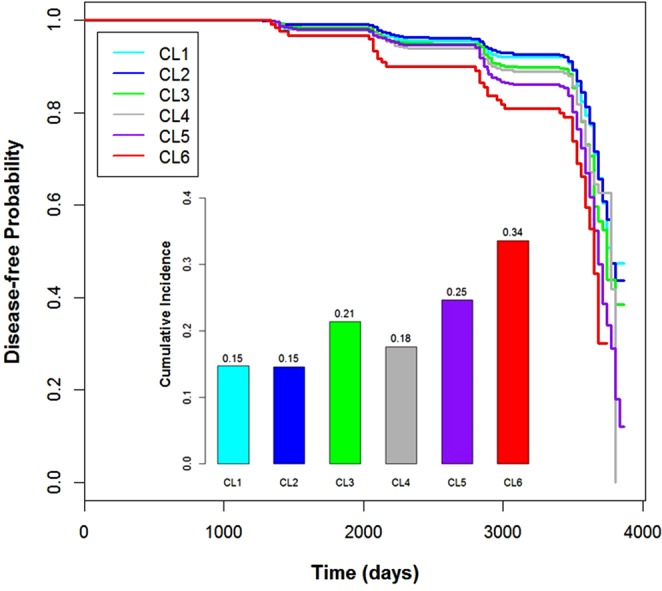
Result of Survival Analysis Using Follow-up of the Discovery Data. A Kaplan–Meier survival curve was fitted based on the disease-free interval from the baseline investigation to the diagnosis of type 2 diabetes or the end of the follow-up. Because the follow-up dates were recoded monthly, we used the first day of the month as follow-up date. The fitted curves of the clusters are located according to the prevalence of the clusters in the discovery data in general, whereas the curve of CL1 (*n* = 1808) is under that of CL2 (*n* = 2937). The bar plot located under the curves indicates the cumulative incidence rates of type 2 diabetes during the follow-up. The cumulative incidence rate for each cluster was estimated as the number of new cases of each cluster during follow-up. As in the discovery data, there was a clear tendency for the cluster-specific incidence rate of type 2 diabetes to correlate with the initial cluster-specific prevalence in general, but the incidence rate of CL3 (*n* = 690) was much higher than that of CL1, CL2, and CL4 (*n* = 807). This was not the case for the prevalence rates in the discovery data. The *n* indicates the initial number of participants in each cluster. The n of CL5 and CL6 is 2050 and 156, respectively. The sharp decrease in survival curves after 3,000 days of follow-up arises from the small number of remaining participants. See Supplementary Results for a detailed explanation.

We performed survival analysis with a disease-free interval and the initial cluster membership as a stratifying factor. Figure 2 shows the Kaplan–Meier plot based on the follow-up data. The log-rank test showed a clear tendency for the cluster-specific incidence rate of type 2 diabetes, which is different between the clusters (*P* < 0.001). The cumulative incidence of type 2 diabetes during follow-up was consistent with the result of survival analysis (Fig. 2). Although the incidence of CL3 was higher than that of CL4, the incidence of other clusters well correlated with prevalence of clusters in general. The results indicated that clusters defined by the risk factors have the implications in the development of future type 2 diabetes. These seem to result from the cluster-specific heterogeneous effect of the risk factors for type 2 diabetes.

### Reproducibility of cluster characteristics in the validation data

After RFC, we built the SVM classification model to determine which cluster each participant belonged to (Supplementary Results for detailed procedure). We applied the cross-sectional validation data to the cluster-discriminating model. As in the discovery data, the prevalence of type 2 diabetes showed a wide discrepancy between clusters (Chi-square test, *P* < 2.2×10^−16^). In general, the predicted clusters in the validation data generally showed the same tendency as the discovery data. The prevalence rates of CL1 (0.04) and CL2 (0.03) were the lowest, as in the discovery data, and the prevalence was higher in CL1 than in CL2, which was opposite to the difference in the discovery data. CL3 and CL4 showed modest prevalence rates (0.11 and 0.10), as in the discovery data, but CL3 had a higher prevalence than CL4. CL5 and CL6 had higher prevalence rates than the other clusters, and the prevalence was higher in CL6 (0.32) than in CL5 (0.17), as in the discovery data. The difference between the highest and lowest cluster prevalence rates (10.7-fold) was greater in the validation data than in the discovery data (4.9-fold).

The unique combination of risk factors for the clusters in the discovery data was reproducible in the cross-sectional validation data, especially for the categorical risk factors (Table 2). The combination of specific categories of the categorical risk factors was consistent in the validation data, except in CL5. In the discovery data, CL5 included participants with HTN and no FHDM. However, in the validation data, CL5 had a few participants with no HTN and FHDM (Table 2). Age and BMI showed differences in their mean values, but the cluster order of the mean age was consistent in the discovery and validation data. The order of mean BMI was inconsistent in CL1–CL4, whereas the order of mean BMI in CL5 and CL6 in discovery data was the same in validation data, and these clusters had the highest mean BMI of all clusters in the both data.

### Differences in biochemical profiles between clusters

We tested whether the biochemical profiles differed according to the cluster membership and diabetes status using two-way analysis of variance (ANOVA). The biochemical profiles included fasting glucose (FG), total cholesterol (TCHOL), triglycerides (TG), and high-density lipoprotein (HDL) levels. For this test, we included participants who had never been diagnosed as having diabetes previously to identify the natural differences in biochemical profiles between clusters in the discovery cohort (*N* = 9,304) and the validation cohort (*N* = 199,827). The FG, TCHOL, TG, and HDL levels showed marginal significance in differences between clusters and in those with diabetes, which were reproducible in the discovery and validation cohorts (*P* < 0.05; Supplementary Fig. 3 and Supplementary Table 1). Moreover, the interactions between cluster membership and diabetes status of all four biomarkers were consistently significant when considering the FG levels in the discovery and validation cohorts (*P* < 0.05; Supplementary Fig. 3).

### Cluster-specific performance in the prediction of type 2 diabetes

Instead of estimating the performance of a single prediction model, we estimated cluster-specific performance of the five risk factors for predicting type 2 diabetes and compared the performance between clusters. In the discovery data, CL2 showed the highest performance (AUC: 0.69, 95% CI: 0.66−0.72) and CL4 showed the lowest (AUC: 0.58, 95% CI: 0.54−0.63). Supplementary Table 2 shows the results when comparing the AUCs between all clusters. As expected, the difference in AUCs between clusters had the highest significance between CL2 and CL4 (*P* < 0.001).

The cluster-specific AUCs in the validation data showed a similar tendency. As in the discovery data, CL2 (AUC: 0.72, 95% CI: 0.72−0.73) and CL3 (AUC: 0.73, 95% CI: 0.72−0.74) showed higher AUCs than those of other clusters and the AUCs between them had no significant difference (*P* = 0.74; Supplementary Table 3). Moreover, CL4 (AUC: 0.58, 95% CI: 0.56−0.59) showed the lowest performance, as in the discovery data. The significance levels of differences in AUCs between clusters were all significant (*P* < 0.001) except for three comparisons (CL2 vs CL3, CL1 vs CL6, and CL4 vs CL5).

**Figure 3:**
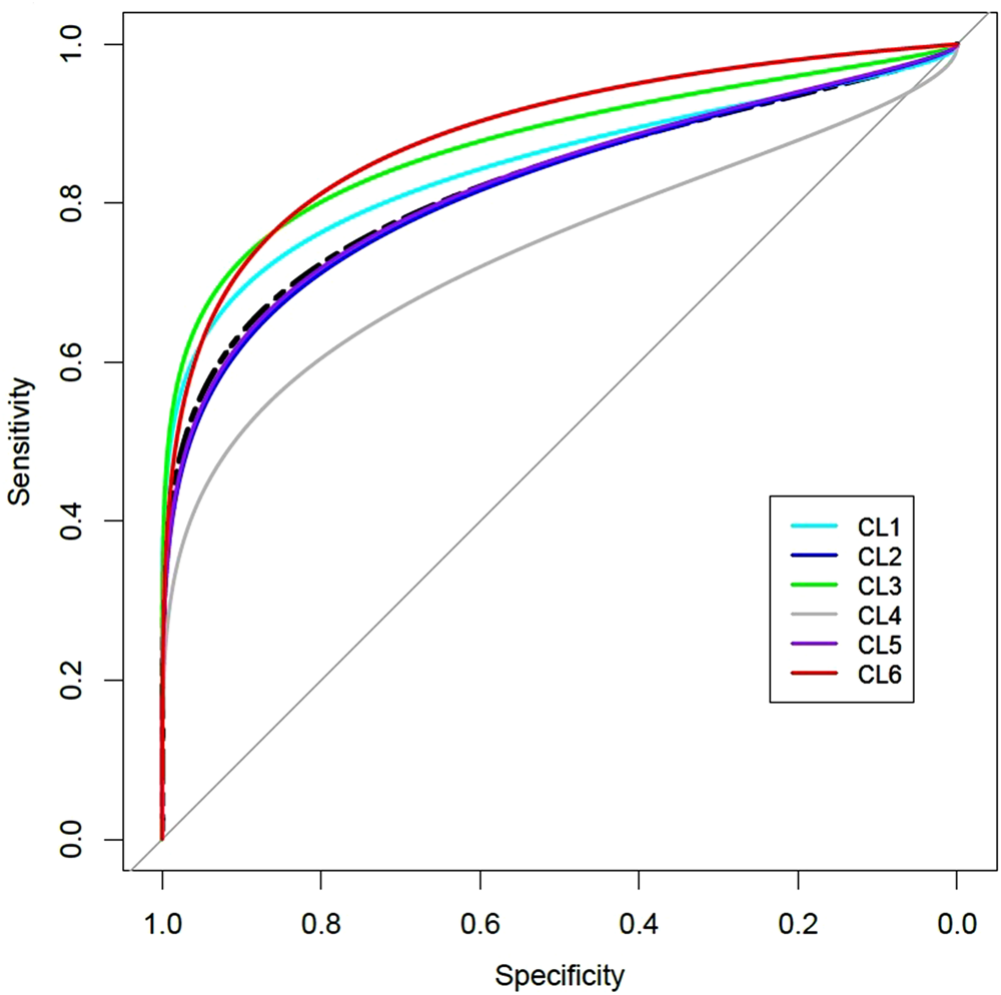
Cluster-specific receive operating curve (ROC) plot of fasting glucose for screening incident diabetes. ROC plots for prediction performance of fasting glucose in the clusters and total population are depicted. The black dotted line is ROC of fasting glucose in the total population (AUC = 0.83). The color of ROCs represents each cluster. The cluster-specific AUC of fasting glucose is the highest in CL6 (AUC = 0.89), while the lowest in CL4 (AUC = 0.74). The AUC of CL1, CL2, CL3, and CL5 is 0.87, 0.82, 0.87 and 0.82, respectively. AUC: area under curve.

We also tested whether the screening performance of the FG on type 2 diabetes differs between clusters of discovery cohort. As shown in Fig. 3 and Supplementary Fig. 5, while AUC of FG with the whole population of discovery cohort is 0.83 (95% CI: 0.81−0.84), the cluster specific AUC varies between clusters. For example, the AUC of CL4 was 0.74 (95% CI: 0.68−0.80), which was the lowest among clusters. The CL6 showed the highest AUC (0.89, 95% CI: 0.83−0.94), and the difference of the two AUC was highly significant (*P* < 0.001). Moreover, the difference between AUC of CL4 and those of the other clusters were all statistically significant (*P* < 0.05, Supplementary Fig. 5).

Finally, we estimated and compared cluster-specific sensitivity and specificity of the FG between clusters of discovery cohort. When we set the predictive threshold of FG from 4.95 to 5.5 mmol/L, the clusters showed a substantial difference in sensitivity and specificity (Supplementary Fig. 6). In general, while sensitivity varied more widely between clusters, the width of the specificity variation was relatively small. CL6 and CL2 showed the highest sensitivity and specificity with all thresholds, respectively.

## Discussion

In this research, we identified population clusters showing difference in the development of type 2 diabetes. The difference in prevalence between clusters was highly significant and reproducible in the validation data. Although the distribution of risk factors showed some discrepancies between the discovery and large-scale validation data, the overall tendency was consistent, especially for the prevalence. Moreover, we found that the prediction performance for type 2 diabetes using well-known predictors (the 5 risk factors and FG) was significantly different between clusters.

We found that the prevalence of type 2 diabetes in the clusters increased as the risk factors became more saturated in the clusters (e.g., older age, higher BMI, or the presence of HTN or FHDM, See Table 2). However, we also found some exceptions that did not show a linear relationship between risk factors and prevalence. Although CL6 had the highest prevalence, the mean age of CL6 was the 4th highest, not the highest in the discovery and validation data. While the average BMI correlated well with the prevalence, CL4 had the lowest and 2nd lowest mean BMI and a moderate prevalence in the discovery and validation data, respectively (Table 2). It is possible that HTN and FHDM, and high mean BMI in CL6 contributed to the high prevalence despite the lower mean age and inclusion of female participants whose risk of type 2 diabetes is generally lower than in males.

Not only the distributions of 5 risk factors and prevalence were different between clusters, the clusters showed the significant difference of biochemical profiles as well (Supplementary Fig. 3 and Supplementary Table 1). The molecules of the biochemical profiles are associated with development of type 2 diabetes^25–28^. Therefore, the result indicates that diabetes-related metabolism might be heterogenous between clusters. Especially, there was significant interaction of FG levels between the diabetes status and cluster membership (Supplementary Fig. 4). The significant interaction indicates that the degree of difference of the FG between diabetes status were different between clusters (Supplementary Fig. 4). While previous research reported significant difference in glycemic profiles between subtypes of type 2 diabetes^29^, our results were obtained from the controls and selected incident diabetes participants who had never been medicated. These findings seem to result from the cluster-specific interaction of the 5 risk factors. The finding suggests that the inherent heterogeneity of clinical features in type 2 diabetes is more complex than is believed at present. From this viewpoint, our results might be applicable to the study of precision medicine that aims to classify subpopulations according to differences in their susceptibility to a particular disease and the biology of that disease^30^. Particularly, our results will be applicable to the novel classification of type 2 diabetes that were suggested in previous research^31^.

Here, we identified significant differences in prediction performance between clusters with different combinations of the five risk factors, which has clinical implications. In both the discovery and validation cohorts, CL2 and CL3 showed higher performance than the other clusters (Supplementary Tables 2 and 3). Moreover, CL4 showed the worst performance consistently. This clearly indicates that prediction models with the five risk factors showed different performance for predicting type 2 diabetes in some population clusters. Indeed, this finding could be observed with a single prediction model. When we built such a model using the complete data and estimated cluster-specific AUCs separately, we identified the same differences in AUCs between clusters (Supplementary Table 4). Given these findings, a single diagnostic or prediction model might be inappropriate for some subpopulations.

The difference in cluster-specific performance of the FG level in predicting type 2 diabetes is another example showing that a single diagnostic model is insufficient for the prediction of type 2 diabetes. As shown in Fig. 3 and Supplementary Fig. 5, the AUC of the FG level in the prediction of incident type 2 diabetes differed significantly between clusters. Consequently, when we applied the same cutoff threshold for the determination of incident diabetes, the clusters showed different sensitivities and specificities (Supplementary Fig. 6). The finding is predictable from the results of two-way ANOVA test of the FG level (Supplementary Fig. 4). If the mean FG in the diabetes group of a cluster is higher than that of the other clusters, the cluster tends to have higher sensitivity with the same cutoff level because diabetes is more prevalent with higher FG levels. Moreover, if the slopes between the mean FG levels of the groups with and without diabetes are parallel, the difference in sensitivity would be maintained. For example, the slopes of CL2, CL4, and CL6 were parallel and their mean FG levels showed clear differences. Consequently, the sensitivity of CL6 was greater than CL2 and CL4, all with different cutoffs, and vice versa for specificities. Moreover, the slopes of CL1 and CL3, which were not parallel with the rest of the clusters, showed different patterns in terms of sensitivity and specificity. These results indicate that for certain subpopulations we should consider different cluster-specific thresholds for the determination of incident diabetes, and not the global threshold. To date, a one-size-fits-all approach has been adopted for construction of prediction models using biomarkers. However, as shown in our results, we should consider the inherent heterogeneity of certain subpopulations in the prediction of disease, at least in cases with type 2 diabetes.

Another implication of the population clusters is that we might be able to develop more cost-effective method in the prevention of type 2 diabetes. It is well-known that the progression from prediabetes to type 2 diabetes can be prevented by the behavioral modification and/or drugs such as metformin^32^. Moreover, application of the preventive methods to the impaired fasting glucose group, which is high risk group for type 2 diabetes, is proven to be cost-effective^33^. Therefore, it is possible that the population clusters might provide an opportunity to identify the subgroup that is more effective with the conventional preventive methods. It is also possible that the clusters would be correlated with the development of diabetes-related complications such as diabetic neuropathy, cerebrovascular accidents, and peripheral vascular disease. Since the biochemical profiles showed clear difference, the incidence of the complications is likely to be different between clusters. These will be investigated in a further study.

In this research, we used machine learning for revealing undiscovered heterogeneity of type 2 diabetes. Machine learning is a field of artificial intelligence in which algorithms can induce knowledge discovery within data^34^. It predicts classes of new instances based on rules or models estimated from the learning examples. Because the sensitive recognition of complex patterns of data is beyond the ability of human perception, machine learning is an appropriate method for such tasks. As shown in our results, machine learning has potential to uncover hidden patterns related to the diagnosis of a disease or to predict disease prognosis automatically.

Our analysis has 2 limitations. First, the discovery data did not include a young-adult generation, especially 20–39 years. Therefore, our cluster-discrimination model does not represent the entire distribution of the risk factors for type 2 diabetes. Second, the incidence of type 2 diabetes was not fully evaluated due to absence of a 2-hour OGTT and HbA_1C_ levels in the cross-sectional validation data. This might have led to underestimation of the prevalence of type 2 diabetes in the cross-sectional validation data.

In summary, we found population clusters with different susceptibilities to type 2 diabetes. The clusters showed different clinical features, and variability of prediction performance for type 2 diabetes. These finding seem to reflect the inherent heterogeneity in the development of type 2 diabetes. We believe that our results will provide opportunities for the development of more precise methods for the management of type 2 diabetes.

## Supplementary information

Supplementary Information
