# Supplementary material for: Identification of novel population clusters with different susceptibilities to type 2 diabetes and their impact on the prediction of diabetes
Source: Sci Rep. 2019 Mar 4;9:3329. doi: 10.1038/s41598-019-40058-y (PMC6399283; doi:10.1038/s41598-019-40058-y)
Supplement: Supplementary file 1 — Supplementary Information [file 41598_2019_40058_MOESM1_ESM.docx]

**Identification of novel population clusters with different susceptibilities to type 2 diabetes and their impact on the prediction of diabetes**

**Seong Beom Cho, M.D., Ph.D**.^*^

Division of Biomedical Informatics, National Research Institute of Health, KCDC

**Sang Cheol Kim, Ph.D**.

Division of Biomedical Informatics, National Research Institute of Health, KCDC

**Myung Guen Chung, M.S.**

Division of Biomedical Informatics, National Research Institute of Health, KCDC

^*^corresponding author

Division of Biomedical Informatics, National Research Institute of Health, KCDC

187, Osongsaengmyeong 2-ro, Osong-eup, Heugndeok-gu, Cheongju-si, Chungcheongbuk-do, 28159, Republic of Korea,

E-mail: sbcho1749@gmail.com,

FAX: 82-43-719-8869

Telephone: 82-719-8850

**Supplementary Method**

**Construction of the SVM model for discriminating clusters in the validation data**

When constructing the cluster-discrimination model for classifying clusters in the validation data, we used a support vector machine (SVM) algorithm. The SVM learns the model for classifying the class labels of instances with training data and predicts the class labels of instance in the test data. Here, the training data was discovery data, and the test data was validation data. After defining the clusters in the discovery data, the cluster numbers were used for class labels and the prediction model for the class labels

The svm function of e1071 R package was used for SVM analysis. In the estimation of the classification model, 2 parameters including gamma and the cost parameter should be adjusted.^1^ We used the tune.svm function to optimize the 2 parameters. The range of gamma was (2^–2^, 2^–1^, 2^0^, 2^1^, and 2^2^) and that of cost parameter was (2^1^, 2^2^, 2^3^, 2^4^, 2^5^, 2^6^, 2^7^, and 2^8^). The assignment of the parameters was suggested by a technical report.^2^ All possible combinations of the 2 parameters were applied by the tune.svm function, and the accuracy of the classification was assessed with 10-fold cross-validation of the original data. The 10-fold cross-validation method is an evaluation method of classification performance without independent validation data, which uses 9/10 of the whole data as the training data and builds the model. After model construction, the remaining 1/10 of the data were used as the test data. The test data were applied to the model without class labeling, and the accuracy of the classification was assessed by comparing the predicted and original class label of the test data. The combinations of the 2 parameters that showed the lowest classification error rate were used to construct the cluster-discrimination model.

**Supplementary Results**

**Filtering of missing values in discovery and validation data**

In the discovery data, seven of 10,030 participants had missing values for the five risk factors. The complete validation data set had 267,411 participants and 52,328 were removed because their age was out of range (<40 or >70 years). The remaining 215,083 participants had no missing values for the five risk factors. Because there were so few missing values, we did not apply any imputation method when carrying out risk factor-based clustering (RFC).

**Determination of cluster numbers**

To determine the cluster number in the discovery data, the chi-square test was applied repeatedly until the −log10(P value) was maximized. As shown in Supplementary Fig. 1, at *k*=2 the result of the chi-square test was already highly significant (P=2.93×10^–34^). As the value of *k* increased by 1, the −log10(P value) increased, but this value decreased when *k*=7. Therefore, we set the number of clusters to 6.

In the RFC, we used the daisy function of the cluster R package for application of the Gower distance. In the coding of categorical variables, the symmetric and asymmetric variables can be assigned according to the same or different weight on the category of variable when determining the dissimilarity of 2 instances. For example, if 1 of the 2 categories of an asymmetric binary variable are identical in a pair of instances (participants in this analysis), the variable has more weight in the computation of dissimilarity between the instances. For symmetric binary variables, the 2 categories are treated equally. The 0/1 coding of hypertension and diabetes status was determined easily because it was obvious that 0 and 1 could be assigned according to the absence and presence of disease, respectively. However, the coding of the sex variable is somewhat controversial, although it is known that males are more susceptible to type 2 diabetes. When we assigned the 0 to females, the –log10(P value) of the determined number of clusters (*k*=3) was 64.16, which was lower than when 0 was assigned to males. Therefore, we used the 0/1 coding system for male/female participants, respectively.

**Construction of the cluster-discrimination model**

The error rate of classification measured with 10-fold cross-validation identified the best combination of the gamma and cost parameters within the predefined combinations. When the gamma = 1 and cost = 128, the error rate was the lowest (=3.99×10^-4^). Therefore, the combination of parameters was applied during construction of the discrimination model, which perfectly discriminated the clusters when the whole discovery data was applied.

**Prevalence of type 2 diabetes as a stopping criterion in the RFC**

In this analysis, we used hierarchical clustering for determination of number of the clusters. Usually, in the cluster analysis, the number of clusters are determined according to the homogeneity within clusters and/or heterogeneity between clusters, which are determined by dissimilarity between samples. However, we used the heterogeneity of diabetes prevalence as a stopping criterion in the RFC, which was measure by Chi-square statistics from cross table of cluster membership and diabetes status. Since the differences between all clusters was not the stopping criterion, there might be a small gap in prevalence between some clusters. However, even in this case, the total heterogeneity of diabetes prevalence can be high because of differences of prevalence between the other clusters. Moreover, even the clusters having a small gap in prevalence, the distribution of the 5 risk factors are different. And, the distribution and prevalence are reproducible in large scale validation cohort. And, the difference of prevalence would implicate difference in pathophysiology of type 2 diabetes development.

**Selection of variables for RFC**

In this analysis, we did not perform any feature selection for RFC because our main hypothesis was that, even with well-known risk factors, a heterogeneous population would show differential susceptibility to type 2 diabetes. Indeed, the five selected risk factors have been identified frequently in previous studies [16]. Moreover, these risk factors had few missing values in the discovery and validation cohorts, which guaranteed larger sample sizes. Therefore, we used these five risk factors for RFC. It is possible that RFC with biochemical profiles that are related to glucose and insulin regulation would reveal heterogeneity among cases of type 2 diabetes other than differences in its prevalence. However, we focused on the heterogeneity of the development of type 2 diabetes in this analysis. Moreover, two-way ANOVA revealed significant differences in biochemical profiles including the levels of FG, triglycerides, total cholesterol, and HDL (Supplementary Table 1). This result implies that the clusters defined by RFC of the five risk factors demonstrate inherent heterogeneity in the biochemical pathophysiology of patients with type 2 diabetes.

**Difference of biochemical profiles between clusters**

We tested whether the biochemical profiles differed according to the cluster membership and diabetes status using two-way analysis of variance (ANOVA). The biochemical profiles included fasting glucose (FG), total cholesterol (TCHOL), triglycerides (TG), and high-density lipoprotein (HDL) levels. For this test, we included participants who had never been diagnosed as having diabetes previously to identify the natural differences in biochemical profiles between clusters in the discovery cohort (*N* = 9,304) and the validation cohort (*N* = 199,827). The FG, TCHOL, TG, and HDL levels showed marginal significance in differences between clusters and in those with diabetes, which were reproducible in the discovery and validation cohorts (*P* < 0.001; Supplementary Fig. 3 and Supplementary Table 1). Moreover, the interactions between cluster membership and diabetes status of all four biomarkers were consistently significant in the discovery and validation cohorts, except for HDL of discovery cohort (*P* < 0.001; Supplementary Table 1).

**Abrupt decline of survival probability within longer follow-up length**

Here, we determined the disease-free interval based on the visiting day of each cohort’s participants during follow-up visits. The estimation of nonparametric Kaplan–Meier survival probability (S(*t_i_*)) was based on the following equation:

$$S\left( t_{i} \right)=\left( 1-\frac{d_{1}}{S_{0}} \right)\times\left( 1-\frac{d_{2}}{S_{1}} \right)\times\left( 1-\frac{d_{3}}{S_{2}} \right)\text{×∙∙∙∙∙×}\left( 1-\frac{d_{i}}{S_{i-1}} \right), i=1, 2,3\ldots.. k,$$

where *t_i_* is the *i-*th time point, and *d* and *S* represent the number of succumbing (with diabetes) and surviving participants (without diabetes), respectively. Therefore, if there was a participant who developed diabetes and had an extreme follow-up duration, the number of surviving participants (*S*) would be smaller. With this condition, even the small number of those developing diabetes could reduce the term$\left( 1-\frac{d_{i}}{S_{i-1}} \right)$, which in turn would reduce the survival probability even to zero in an extreme case. The survival curve of CL4 is a typical example of such an extreme case. In CL4, only one participant had the longest follow-up time (3,804 days) and developed diabetes. Moreover, there was only one surviving participant without diabetes at the previous time point. Therefore, the survival probability became zero (see Fig. 2). In the other clusters, few participants had extreme follow-up durations. Thus, even if there were a few participants who developed diabetes, the survival probability would show an abrupt decline.

**Supplementary References**

**1.** Meyer D, Dimitriadou E, Hornik K, Weingessel A, Leisch F. e1071: Misc functions of the Department of Statistics, Probability Theory Group (Formerly: E1071). R package version 1.6-7, 2015. (Accessed August 5, 2015 at https://CRAN.R-project.org/package=e1071)

**2.** Hsu CW, Chang CC, Lin CJ. A practical guide to support vector classification, 2003. (Accessed October 15, 2016 at http://www.csie.ntu.edu.tw/~cjlin/papers/guide/guide.pdf)

**3.** E. Mooi and M. Sarstedt, A Concise Guide to Market Research, DOI 10.1007/978-3-642-12541-6_9, Springer-Verlag Berlin Heidelberg 2011

**Supplementary figure legends**

**Supplementary Figure 1. Degree of Heterogeneity in the Prevalence between the Clusters in the Discovery Data.**

The −log10(P values) from the chi-square test of heterogeneity between clusters are depicted. At *k*=2, the degree of heterogeneity was high (P=2.93×10^–34^). As the value of *k* increased, the −log10(P value) increased. The −log10(P values) for *k*=3 and *k=*4 were similar, although the value was slightly higher at *k*=4 (63.08) than at *k*=3 (62.86).

**Supplementary Figure 2. Cluster Dendrogram of the Discovery Data by RFC.**

The participants in each cluster are delineated by rectangles of different colors. CL refers to the cluster numbers (e.g., CL1 = cluster 1). The clusters are numbered incrementally by ascending order of the cluster-specific prevalence of type 2 diabetes.

**Supplementary Figure 3. Box plots for cluster-specific means of biochemical profiles in nondiabetes and diabetes group.**

The cluster-wise means of FG, TC, TG, HDL in non-diabetes and diabetes groups are plotted. FG: fasting glucose, TC: total cholesterol, TG: triglyceride, HDL: high-density lipoprotein

**Supplementary Figure 4. Cluster-specific difference of fasting glucose level between diabetes and nondiabetes groups.**

The means of diabetes and nondiabetes group in each cluster are plotted. The slopes between the means of diabetes and nondiabetes group were parallel in CL2, CL4, CL5 and CL6. The slopes of CL1 and CL3 showed deviation from the other slopes.

**Supplementary Figure 5. Difference of AUCs between clusters.**

The numbers on the top of bar plots are AUC values. The P values are presented on the lines between clusters, which comes from comparison of AUC of CL4 and the other clusters using roc.test in pROC R package.

**Supplementary Figure 6. Sensitivity and specificity of fasting glucose in each cluster.**

Sensitivities and specificities for prediction of incident diabetes are presented according to the different thresholds. The colors indicate corresponding clusters.

**Supplementary Fig. 1**


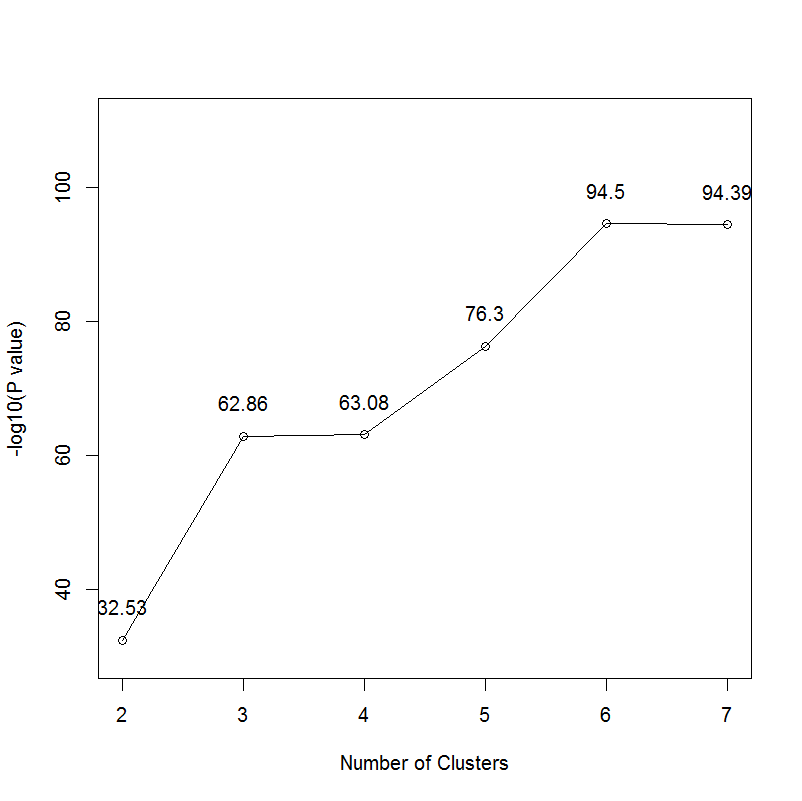


**Supplementary Fig. 2**


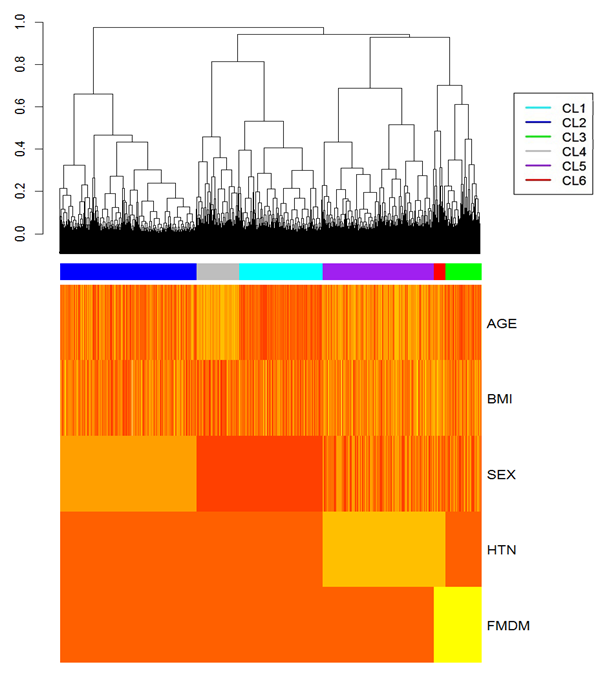


**Supplementary Fig. 3**

**
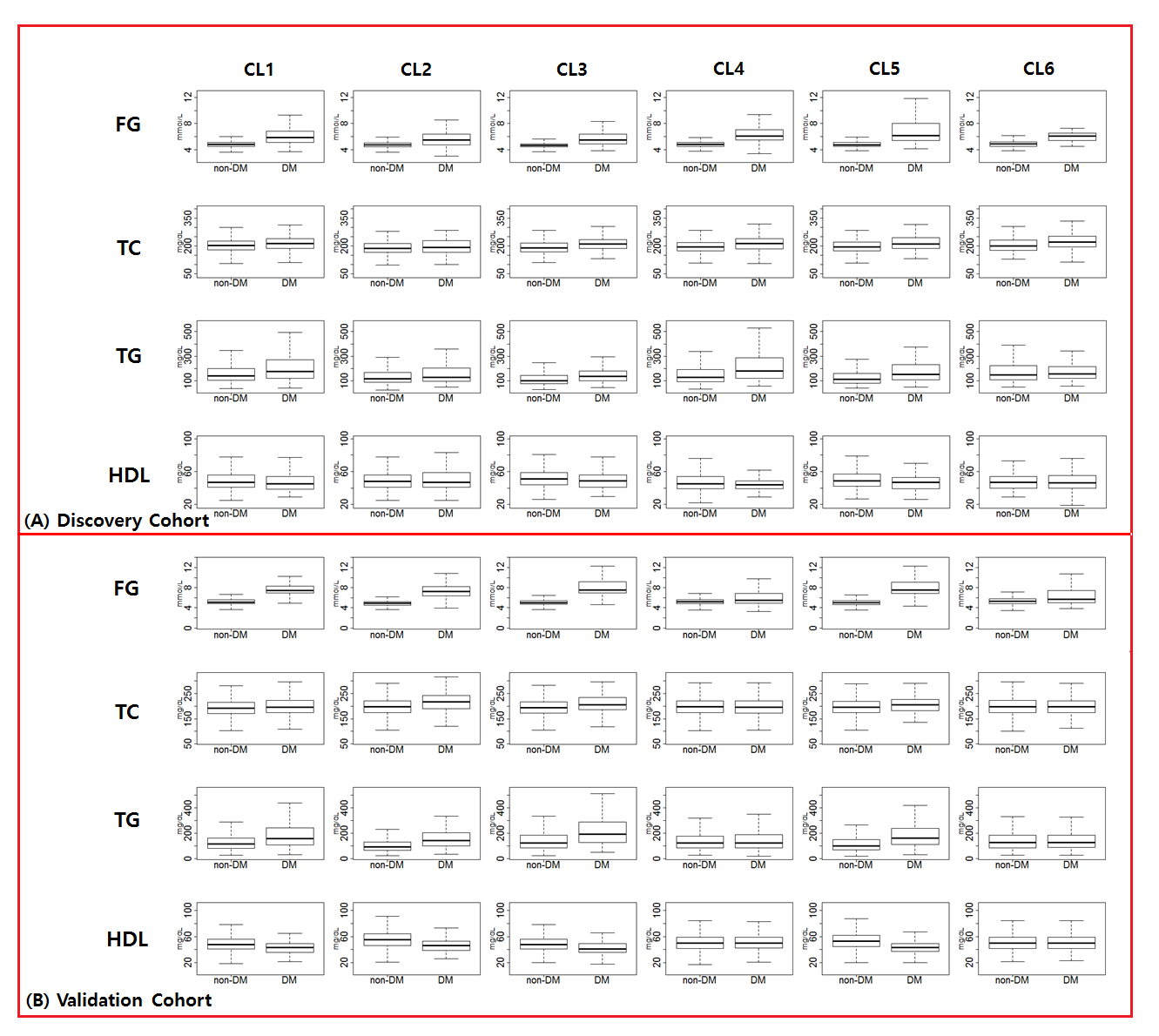
**

**Supplementary Fig. 4**

**
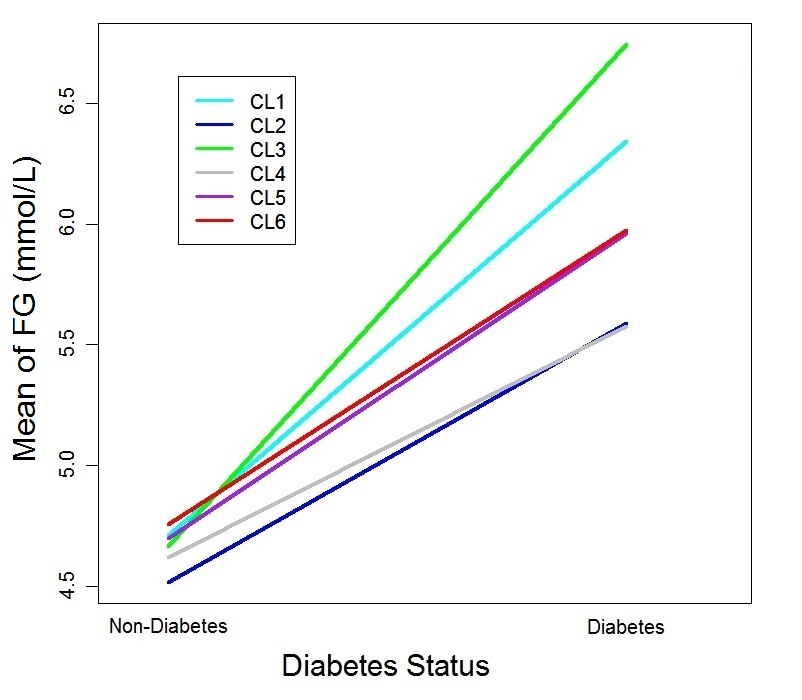
**

**Supplementary Fig. 5**


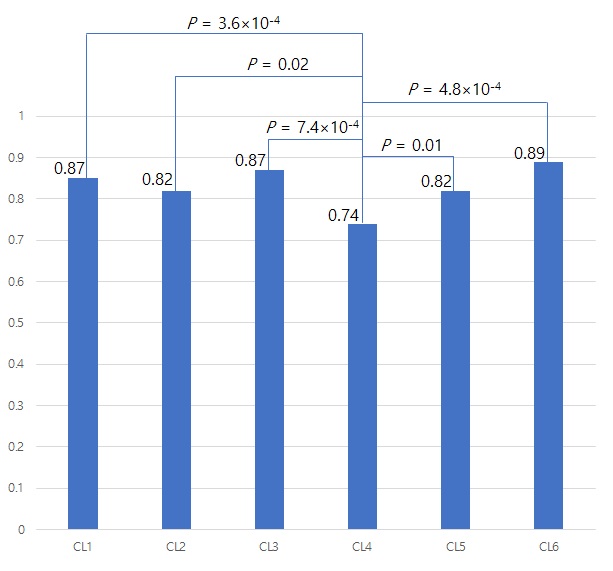


**Supplementary Fig. 6**

**Supplementary Tables**

**Supplementary Table 1. Result of two-way ANOVA of FG, TC, TG and HDL in discovery and validation cohort**

|  | | Discovery (*N* = 9,304) | | | | | Validation (*N* = 199,827) | | | | |
| --- | --- | --- | --- | --- | --- | --- | --- | --- | --- | --- | --- |
|  |  | DF | SS | MS | F value | *P* | DF | SS | MS | F value | *P* |
| FG | cl.ind | 5 | 66051.55 | 13210.31 | 69.44846 | 1.54E-71 | 5 | 68997565 | 13799513 | 23.81112 | 5.06E-24 |
|  | DM | 1 | 631196.7 | 631196.7 | 3318.29 | 0 | 1 | 3.81E+08 | 3.81E+08 | 656.8579 | 1.27E-144 |
|  | cl.ind:DM | 5 | 36630.18 | 7326.037 | 38.51401 | 2.86E-39 | 5 | 2.14E+08 | 42748146 | 73.76212 | 1.85E-77 |
|  | Residuals | 9292 | 1767500 | 190.2174 | NA | NA | 191704 | 1.11E+11 | 579540.6 | NA | NA |
| TC | cl.ind | 5 | 252158.3 | 50431.66 | 39.36829 | 3.65E-40 | 5 | 753807.6 | 150761.5 | 120.2303 | 1.82E-127 |
|  | DM | 1 | 147066.3 | 147066.3 | 114.8038 | 1.24E-26 | 1 | 27586.5 | 27586.5 | 21.99985 | 2.73E-06 |
|  | cl.ind:DM | 5 | 28999.62 | 5799.923 | 4.527574 | 0.0004 | 5 | 175290.8 | 35058.15 | 27.9584 | 2.03E-28 |
|  | Residuals | 9292 | 11903259 | 1281.022 | NA | NA | 191674 | 2.4E+08 | 1253.94 | NA | NA |
| TG | cl.ind | 5 | 4568846 | 913769.3 | 85.84813 | 1.69E-88 | 5 | 64111828 | 12822366 | 1592.47 | 0 |
|  | DM | 1 | 1747523 | 1747523 | 164.1788 | 2.86E-37 | 1 | 2462753 | 2462753 | 305.8609 | 1.97E-68 |
|  | cl.ind:DM | 5 | 253405.2 | 50681.05 | 4.761457 | 0.000239 | 5 | 3394792 | 678958.4 | 84.32304 | 8.17E-89 |
|  | Residuals | 9292 | 98904242 | 10644.02 | NA | NA | 191378 | 1.54E+09 | 8051.873 | NA | NA |
| HDL | cl.ind | 5 | 30549.07 | 6109.814 | 44.10048 | 4.06E-45 | 5 | 1319464 | 263892.7 | 1616.544 | 0 |
|  | DM | 1 | 2244.599 | 2244.599 | 16.20145 | 5.74E-05 | 1 | 11519.9 | 11519.9 | 70.56817 | 4.48E-17 |
|  | cl.ind:DM | 5 | 1178.137 | 235.6274 | 1.700752 | 0.130689 | 5 | 47991.17 | 9598.235 | 58.79648 | 2.20E-61 |
|  | Residuals | 9292 | 1287342 | 138.543 | NA | NA | 191670 | 31289178 | 163.245 | NA | NA |

cl.ind : cluster indication, DM: diabetes, cl.ind:DM: interaction between cl.ind and DM, SS: sum of square, MS: mean square, DF: degree of freedom, *P*: p value, FG: fasting glucose, TC: total cholesterol, TG: triglyceride, HDL: high-density lipoprotein. The total number of participants without missing value is DF of residuals + 12.

**Supplementary Table 2. Result of comparison of prediction performance between clusters of discovery cohort.** The value between parentheses in the row and column of table is area under curve (AUC) estimated from data of each cluster. The values within table are *P* values that indicate significance of difference between the AUCs of two clusters. The significance was estimated from the roc.test function of the pROC R package [21].

|  | CL1 (0.62) | CL2 (0.69) | CL3 (0.66) | CL4 (0.58) | CL5 (0.63) | CL6 (0.67) |
| --- | --- | --- | --- | --- | --- | --- |
| CL1 (0.62) | NA | 0.011 | 0.156 | 0.261 | 0.698 | 0.212 |
| CL2 (0.69) | NA | NA | 0.371 | 1.396E-04 | 0.003 | 0.589 |
| CL3 (0.66) | NA | NA | NA | 0.012 | 0.178 | 0.904 |
| CL4 (0.58) | NA | NA | NA | NA | 0.079 | 0.033 |
| CL5 (0.63) | NA | NA | NA | NA | NA | 0.263 |
| CL6 (0.67) | NA | NA | NA | NA | NA | NA |

CL: cluster

**Supplementary Table 3. Result of comparison of prediction performance between clusters of validation cohort.** All notations were the same with those of Supplementary Table 2.

|  | CL1 (0.62) | CL2 (0.72) | CL3 (0.73) | CL4 (0.58) | CL5 (0.58) | CL6 (0.62) |
| --- | --- | --- | --- | --- | --- | --- |
| CL1 (0.62) | NA | 6.56E-25 | 3.42E-26 | 2.05E-05 | 1.23E-06 | 0.797 |
| CL2 (0.72) | NA | NA | 0.736 | 4.14E-72 | 1.51E-140 | 8.81E-43 |
| CL3 (0.73) | NA | NA | NA | 2.73E-75 | 8.72E-149 | 2.69E-45 |
| CL4 (0.58) | NA | NA | NA | NA | 0.823 | 1.03E-06 |
| CL5 (0.58) | NA | NA | NA | NA | NA | 6.79E-10 |
| CL6 (0.62) | NA | NA | NA | NA | NA | NA |

CL: cluster

**Supplementary Table 4. Cluster-specific prediction performance with model estimated from whole data.** In discovery and validation cohort, the prediction model for type 2 diabetes using the 5 risk factors are constructed with whole data, and diabetes status was predicted. Then, AUC are determined on the predicted diabetes status of the participants in each cluster.

| Cluster | Discovery cohort | | Validation cohort | |
| --- | --- | --- | --- | --- |
|  | **AUC** | **95% CI** | **AUC** | 95% CI |
| CL1 | 0.616 | 0.572 − 0.660 | 0.623 | 0.605 − 0.640 |
| CL2 | 0.689 | 0.658 − 0.719 | 0.724 | 0.714 − 0.733 |
| CL3 | 0.660 | 0.615 − 0.704 | 0.726 | 0.717 − 0.735 |
| CL4 | 0.579 | 0.536 − 0.622 | 0.575 | 0.561 − 0.588 |
| CL5 | 0.624 | 0.599 − 0.649 | 0.576 | 0.569 − 0.582 |
| CL6 | 0.669 | 0.605 − 0.734 | 0.616 | 0.604 − 0.627 |

AUC: area under curve, CI: confidence interval
